# Materials-Driven Advancements in Chipless Radio-Frequency Identification and Antenna Technologies

**DOI:** 10.3390/s25092867

**Published:** 2025-05-01

**Authors:** Hafsa Anam, Syed Muzahir Abbas, Iain B. Collings, Subhas Mukhopadhyay

**Affiliations:** School of Engineering, Faculty of Science and Engineering, Macquarie, Sydney, NSW 2109, Australia; syed.abbas@mq.edu.au (S.M.A.); iain.collings@mq.edu.au (I.B.C.); subhas.mukhopadhyay@mq.edu.au (S.M.)

**Keywords:** chipless, RF tags, passive RFID tags, low cost, CRFID, WSN, auto-ID, UHF RFID, IoT

## Abstract

This article presents a comprehensive analysis of the technical characteristics of advanced versatile materials used in chipless radio-frequency identification (RFID) tags and antennas. The focus is on materials that are used as radiators and substrates. Crucial aspects include flexibility, weight, size, gain, environmental sustainability, efficiency, fabrication time and type, and cost. A comprehensive set of tables are presented that summarize and compare material properties. The materials include flexible high-tech ink substances, graphene, and liquid crystals, as well as metamaterials which possess properties that allow for an increased bandwidth. Printing techniques are discussed for high-performance high-resolution fabricated tags. This paper contributes by systematically comparing emerging materials for chipless RFID tags, highlighting their impact on performance and sustainability. It also provides practical guidance for material selection and fabrication techniques to enable next-generation wireless applications. It presents a broad understanding of various materials and their use. The paper provides direction for the deployment and utilization of inexpensive passive chipless RFID tags in future intelligent wireless networks. The advancement of chipless RFID is largely driven by the development of innovative materials, especially in the realm of advanced materials and smart materials, which enable the creation of more cost-effective, flexible, and scalable RFID systems.

## 1. Introduction

The first single-bit radio-frequency identification (RFID) tag was developed in the 1960s [1], used as an anti-theft technology, and during the 1960s and 1970s RFID technology was used in military applications involving safety and security [2,3]. Currently, RFID technology is extensively utilized in versatile fields for extensive applications [4,5]. RFID tags are categorized into three categories: passive, semi-passive, and active tags. The differentiator is the presence or absence of a battery or other power source. All categories are embedded with an integrated chip (IC). The power source, read range, type, signal range, data storage and transmission, cost, lifespan, and application are major variants for these categories. Almost one half of RFID deployment around the world depends on battery-free RFID tags, and currently passive RF tags are a roughly USD 5 billion industry, with a vigorous upward trend predicted over the coming years [6].

**Figure 1:**
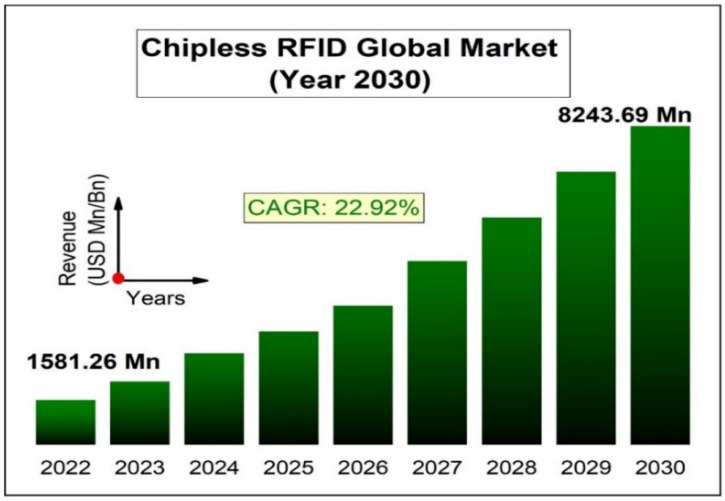
Global chipless RFID market (2030) [6].

**Figure 2:**
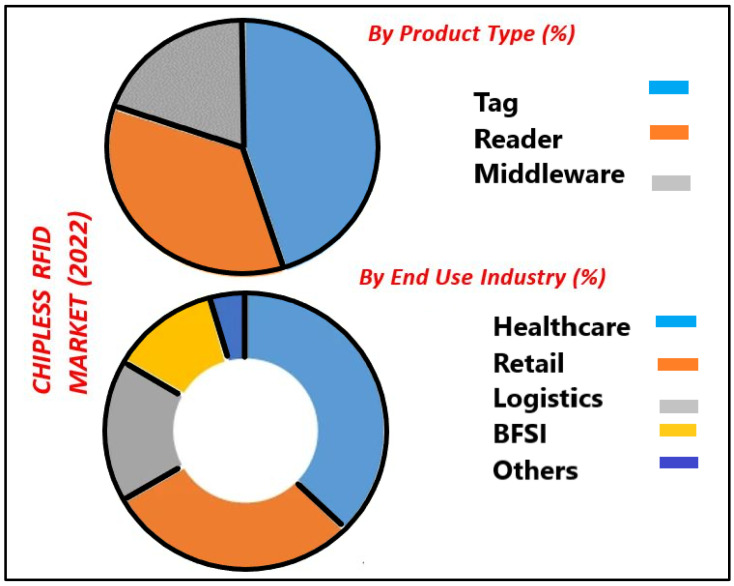
Chipless RFID market in 2022 [7].

In the 1980s, the concept of a passive chipless RFID (CRFID) tag came into being [3]. CRFID is a wireless data capturing and monitoring technology that has more recently found widespread application, including as a preferred technology to traditional bar-codes for automated identification [1]. CRFID is a class of RFID that does not have an integrated IC or a battery. CRFID offers contactless identification capabilities as well as sensing and data harvesting capabilities [2]. CRFID is particularly suited to applications involving low-cost items/equipment, where more complex processor-based sensors are infeasible. As with passive RFID, they also use incident RF wave power to scatter back coded information. Figure 1 shows the predicted global CRFID market for the year 2030 [6], and the CRFID market by product type and industry dependency is shown in Figure 2 [7]. A cost of less than 1 cent is possible for such tags [8].

**Figure 3:**
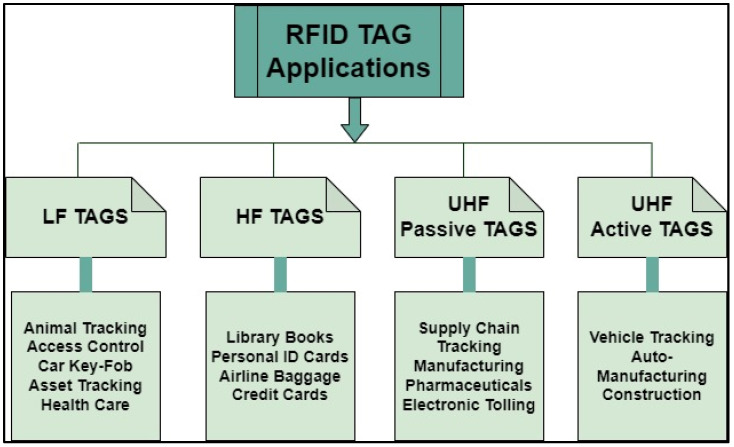
RFID tag applications.

The frequency range of operations is very important. Low-frequency (LF) tags operate at low frequencies (30 kHz to 300 kHz) and have a short read range compared to UHF and high-frequency (HF) tags. HF tags operate at (3 MHz to 30 MHz) for larger read ranges [2]. Any RFID frequency range can be used for passive tags. LF tags are not suitable for some applications because of their low data capacity. On the other hand, high-frequency (HF) passive tags offer notable advantages, including extended read range, improved data transmission rates, and compact physical dimensions. However, these benefits are typically not achieved simultaneously within a single implementation. In contrast, ultra-high-frequency (UHF) passive tags represent a more comprehensive solution, as they are capable of concurrently delivering long read ranges, high data rates, and compact form factors [2]. A few applications of RFID tags classified as LF, HF, and UHF RF identification tags are depicted in Figure 3.

**Figure 4:**
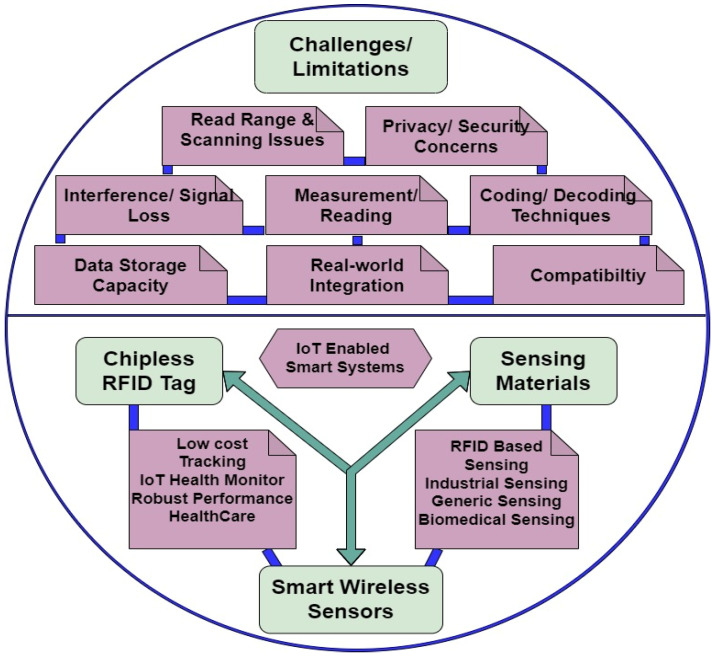
Limitations and objectives of chipless RFID technology.

In the last few years, there has been great interest in embedding sensing materials in pre-existing RFID technology, particularly with the growth of the Internet of Things (IoT). Applications that require both sensing and identification include the remote sensing of moisture [9,10], temperature [11,12], and strain [13,14]. Other examples include supply chain management [15,16,17], smart card applications, automobile immobilization, and animal identification. CRFID development has recently been focused on aggregation of sensing attributes in identification tags, to cope with the growing demands of smart sensor networks. Consequently, increased emphasis has been placed on the selection and engineering of materials utilized in CRFID design. The integration of advanced functionalities into RFID tags necessitates precise material design and optimization to ensure consistent and reliable performance under varying environmental and operational conditions. Figure 4 illustrates some of the challenges of developing CRFID technology across all these application areas. Chipless radio-frequency identification (RFID) technology faces several real-world implementation challenges. Readability on various objects, misalignment between reader antenna and tag, cost and printability, real-time testing, real-time deployment challenges, and misalignment are a few real-world issues of the technology. Overcoming these challenges is essential for the widespread adoption and success of chipless RFID technology [18,19,20].

The main driving force for the evolution of chipless RFID is the development of advanced materials that can replicate the function of a traditional RFID chip. Metamaterials are engineered materials designed to have properties that may not be found in naturally occurring substances. They can manipulate electromagnetic waves in ways that traditional materials cannot, which is crucial for chipless RFID. They have frequency-dependent behavior, enhanced signal interaction, and allow for the miniaturization of RFID tags, making them smaller, lighter, and potentially less expensive to produce. Secondly, conductive polymers and carbon nanotubes make it possible to develop flexible and stretchable chipless RFID tags. These materials can conform to irregular surfaces, making RFID tags applicable in a wider range of environments (e.g., wearable electronics, curved surfaces, and packaging). Carbon nanotubes exhibit excellent electrical conductivity and can be used to create highly sensitive RFID sensors that are crucial for chipless RFID systems. Third, printed electronics, including conductive inks and flexible substrates, enable low-cost mass production of chipless RFID tags. This can be especially useful for applications like product labeling, logistics, and inventory management, where cost is a critical factor. Printed electronics can be integrated into other devices or packaging materials, enabling RFID to become an integral part of larger systems, such as smart packaging or wearable devices. Lastly, dielectric materials are used to modify the resonant frequencies of RFID tags in chipless systems. By using materials with specific dielectric properties, manufacturers can fine-tune the frequency at which the tags resonate, ensuring they operate efficiently in a broad range of environmental conditions. This paper discusses how the intrinsic properties of materials, such as conductivity, dielectric behavior, and mechanical robustness, directly influence the functionality and scalability of RFID systems. The role of materials in enabling improved device performance, such as enhanced signal sensitivity, flexibility, environmental stability, and cost-effectiveness, is very important.

**Figure 5:**
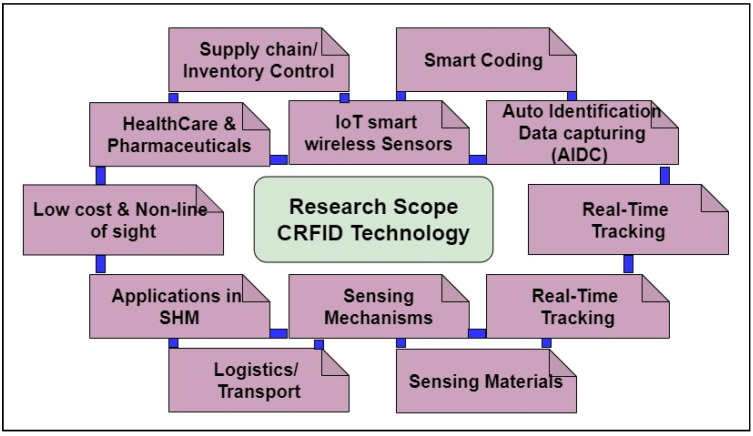
Research scope of chipless RFID technology.

This review article provides an analysis of versatile materials deployed as substrates and radiators for CRFID. Figure 5 provides a visualization of the current scope of CRFID technology. Recent RFID technology developments focusing on sensing solutions can be found in [21,22,23], and some specific CRFID tag designs have been discussed in [24], while application-based reviews can also be found in [25]. The specific topic of surface acoustic wave (SAW)-based solutions is addressed in [26].

This paper presents CRFID technology from the perspective of materials used for tag design and implementation. Some materials are flexible, others are rigid. Advanced materials with promising features and challenging attributes are discussed. Textile and liquid crystal material properties are also discussed, as well as printable materials.

The general distribution of this review paper is organized in a way that the article is split into two halves: one is radiators and the other is substrate materials. Section 2 gives an overview of the working principles of chipless RFID, Section 3 depicts chipless RFID materials being utilized as substrates. Section 4 introduces detailed analysis of materials being used as radiators. Finally, in Section 5 the proposed article is concluded.

## 2. Working Principle of CRFID Tags

**Figure 6:**
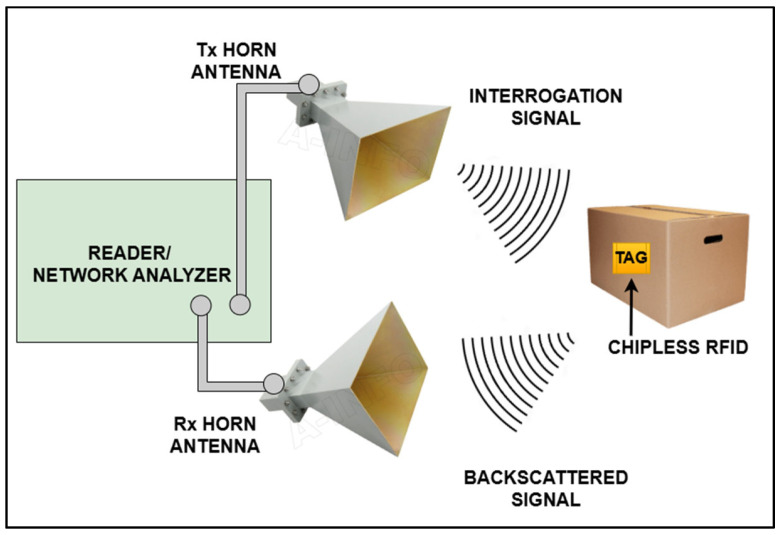
Typical laboratory configuration of the RFID tag characterization system.

**Figure 7:**
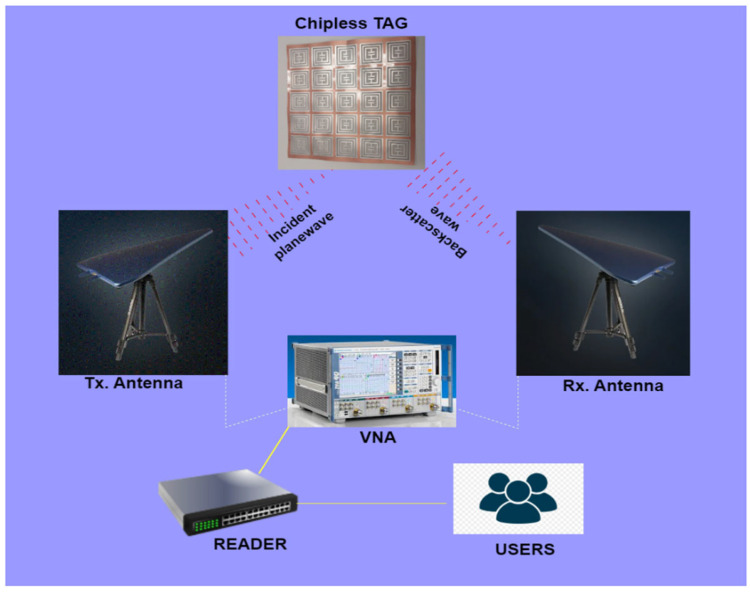
Architecture of CRFID sensor system [27].

The components of a chipless RFID experimental setup are a vector network analyzer (VNA), transmitter and receiver horn antennas, a chip-free tag, an RFID reader, a signal processing unit, and a server. The experimental configuration is shown in Figure 6. The tag is mounted at a far-field distance from the antennas. The tag is read by readers and multiple software tools are used, i.e., CST Studio Suite 2020 (Computer Simulation Tool), HFSS (High Frequency Selective Surfaces), Matlab, RFID Middleware, and various reader-specific software. This software is used to manage and process data from multiple RFID readers. Middleware is typically used when multiple RFID readers are involved, and the data need to be processed or passed to other systems. Features involved in the reader software are real-time data processing, tag tracking, integration with databases, and event-driven workflows. Chipless RFID tags operate via ‘backscattering’ phenomena [2]. Incident electromagnetic (EM) waves from transmitter antennas fall on the surface of the tag. Current is induced at surface of the tag and data are encoded in the EM wave. The data-capturing wave then backscatters towards the reader’s VNA, where it is read and decoded at the reader end. The basic architecture of a sensor RFID tag is shown in Figure 7 [27]. Here, the sensor tag is deployed over the surface of the material under test (MUT), set at a far-field distance from the reader’s VNA. The data captured by the sensor tags are stored and mapped at the back-end sever and sense the required state of the object. The reader controls/sends electromagnetic waves, which are then reflected by the chipless RFID tags. Subsequently, the reader receives and deciphers these reflected signals. The antenna, which is linked to the RFID reader, is tasked with sending out electromagnetic waves to the chipless RFID tags and then receiving the signals that are reflected. The signal processing unit is tasked with handling the signals that the RFID reader receives. It interprets the distinct reflection patterns from the chipless RFID tags and converts them into useful data. The interpreted data from the chipless RFID tags are usually stored and overseen in a database or server. This facilitates the real-time monitoring and administration of the tagged items. The user interface/application refers to the RFID system’s user interface, which enables users to observe and control the tagged items in real time. The elements and their operations can differ based on the exact setup of the chipless RFID system and desired application.

Power utilization is efficient in chipless RFID tags because the data transmission occurs without any power input via backscattering phenomena. The tag may be designed in a variety of unique shapes and dimensions, each capable of generating a distinct bit-code corresponding to a specific data capacity. Diverse tag responses can be engineered by configuring the frequency response output through combinations of versatile resonators. These resonators exhibit characteristic scattering behaviors or resonance frequencies, which are directly influenced by the specific geometry of the backscattering tag. Chipless RFID tags encapsulate data-capturing functionality via utilization of their unique structural designs. The structure can be either an antenna, a microstrip-line, or resonator-based. The latter category, ‘resonator based’, formulates a resonator-based chipless RFID tag structure that yields an identification code via backscattering phenomena. Furthermore, chipless RFID systems can be broadly categorized into spatial-domain, time-domain, and frequency-domain approaches. Among these, frequency-domain-based chipless RFID systems are of particular interest due to their significantly higher data capacity relative to other techniques. In addition, they typically offer superior read range performance. While time-domain systems encode information along the temporal axis, frequency-domain systems utilize the frequency spectrum for data representation.

**Figure 8:**
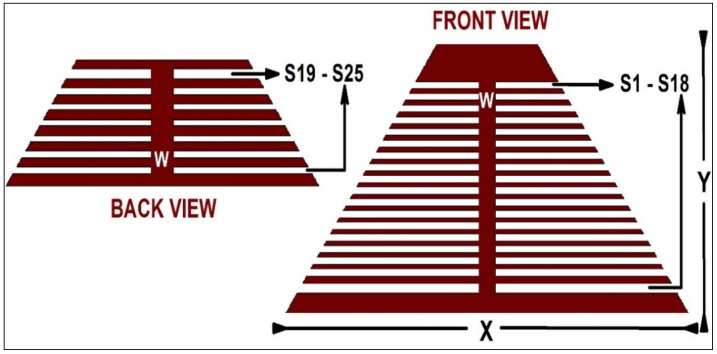
A 25-bit chipless RFID tag design [31].

**Figure 9:**
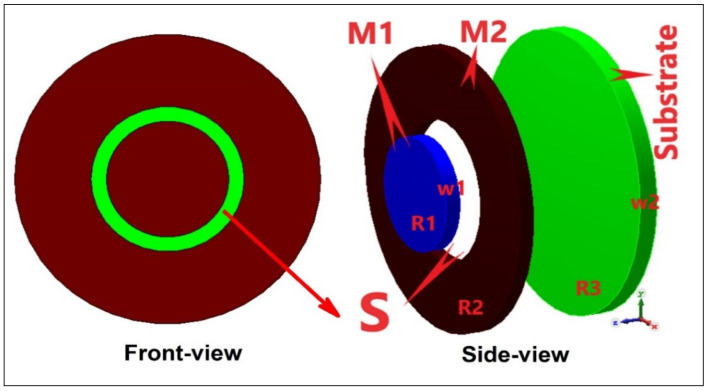
A 1-bit chipless RFID circular tag design [28].

**Figure 10:**
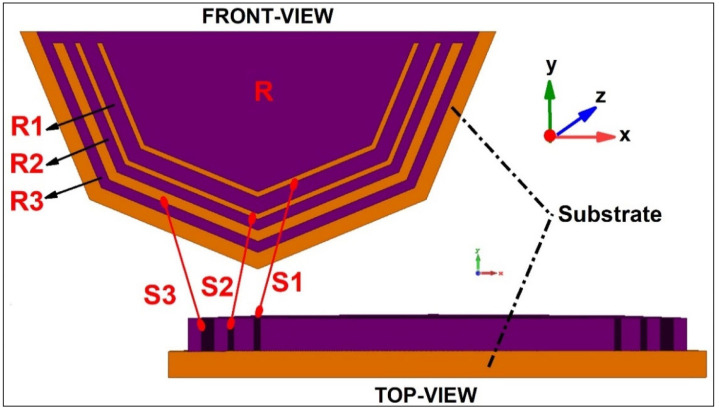
A 3-bit chipless RFID tag for recycle unit management [29].

The RFID tag design requirements that need to be considered when designing an RFID system/tag normally depends upon the application for which the tag is going to be operational. Cost, area/dimensions, frequency of operation, read range, reliability, security, and application, along with mobility, are a few important points to be monitored while designing the tag [2]. The initial step in the design process involves collecting the requirements and establishing the specifications for the RFID tag. These requirements encompass aspects such as performance, read range, reader, quality, data capacity, and the physical layout of the tag. The tag design is dependent upon the lengths and radii of the etched slots. A chipless RFID tag designed for 25 bits is shown in Figure 8. In the tag design, it can be that each slot is of a different length, resonating at a different frequency. The shortest slots resonate at the largest frequency (‘LSB’; least significant bit), whereas the ‘MSB’ (most significant bit) is a dip acquired at the smallest frequency. The resonant frequency of each slot can be found using Equation (1) [8], dependent upon the length of the slot.

(1) $$f_{r}=\frac{c}{2L}\sqrt{\frac{2}{{Ɛ}_{r}+1}}$$

where ‘c’ is the speed of light (3 × 10^8^ m/s), ‘L’ is the length of the resonating slot, and ${‘Ɛ}_{r}’$ is the permittivity of the dielectric used. For a circular slot design, the resonance frequency is radius dependent. One such tag is shown in Figure 9 [28]. Figure 10 also shows a chipless RFID Tag with 3 bits dedicated towards a recycling unit application [29]. Larger-radius slots resonate at a smaller frequency, whereas smaller-radius slots correspond to a higher-frequency notch. The resonance frequency of circular tags is dependent upon the radius of the slots, as given in Equation (2) [30]:

(2) $$f_{r}=\frac{c}{2\pi R}\sqrt{\frac{2}{{Ɛ}_{r}+1}}$$

Here, ‘R’ is the radius of the slot of the designed tag. Moreover, the radar cross-section (RCS) response of the fabricated tags is typically measured via the standard measurement formula [8], given by Equation (3):

(3) $$\sigma^{tag}={\left[\frac{s_{11}^{tag}-s_{11}^{i}}{s_{11}^{\gamma ef}-s_{11}^{i}}\right]}^{2}\cdot \sigma^{\gamma \mathrm{e}f}$$

where $\sigma^{tag}$ is the RCS value retrieved from the proposed chipless tag, $\sigma^{\gamma \mathrm{e}f}$ is the known reference RCS value, $s_{11}^{tag}$ is the measured S11 value of the proposed tag, $s_{11}^{\gamma ef}$ is the measured value of the S-parameter as a reference, and $s_{11}^{i}$ is the tagless value acquired via measurement in an isolated setup; S11 is the reflection coefficient.

The following equations illustrate the core principles of antenna theory and demonstrate their application in the analysis, design, and measurement of antennas [32]. The major categories of antennas, each serving specific functions, are loops, arrays, broadband antennas, reflector antennas, linear dipoles, horns, and aperture antennas. One of the very important parameters of an antenna is the ‘gain’, defined as follows in Equations (4) and (5) [32]:

(4) $$Gain=4\pi \;\frac{radiation\;intensity\;}{total\;input\;power}$$

(5) $$G=4\pi \frac{U\;\left(\theta ,\;ɸ\right)}{P_{in}}\;$$

where $‘P_{in}’$ is the total input power. The total radiating power is given by Equation (6) [32]:

(6) $$P_{rad}=e_{cd}\;P_{in}$$

where $‘e_{cd}’$ is the radiation efficiency of antenna. The Friis transmission equation is given by Equation (7) [32]:

(7) $$\frac{P_{r}}{P_{t}}=e_{t}\;e_{r}\;\frac{\lambda^{2}\;D_{t}\;\left(\theta_{t}\;,\;\;{ɸ}_{t}\right)\;D_{r}\;\left(\theta_{r}\;,\;\;{ɸ}_{r}\right)\;}{{\left(4\pi R\right)}^{2}}$$

where $‘D_{t}\left(\theta_{t},{ɸ}_{t}\right)’$ is the directivity of the transmitting antennas, $‘D_{r}\left(\theta_{r},{ɸ}_{r}\right)’$ is the receiver antenna’s directivity, and $‘e_{t}’$ and ‘$e_{r}’$ are the transmitting and receiving antennas’ radiation efficiency.

## 3. Substrate Material

**Figure 11:**
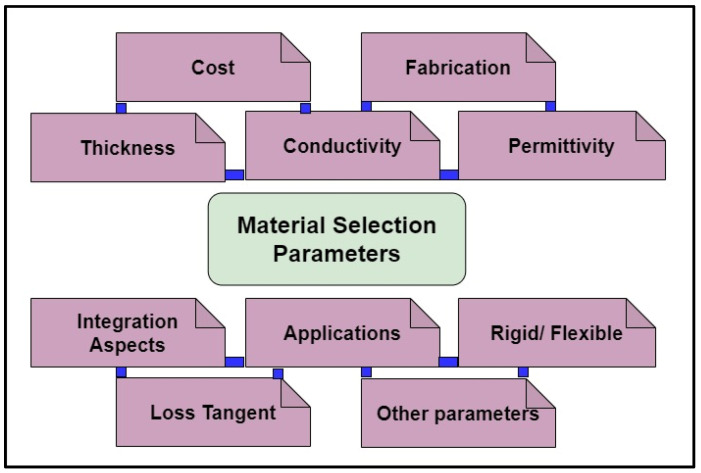
Material selection parameters.

Modern, smart, easy-to-deploy materials are in demand, with intensive development of wireless sensor networks (WSNs) and IoT. Researchers are looking forward to coming up with smart identification/sensing models to communicate/link with an encompassing environment via intelligent outputs. The need for the future is to deploy such smart and flexible sensors in artificial intelligence (AI)-based IoT system, that have the capability to be incorporated and linked into nearly everything. Electronic intelligence is a near-future technique to be acquired via low-cost flexible and plastic materials that will dramatically change working efficiency/output along with the cost of systems [33]. Materials for a transponder/antenna are selected depending upon various parameters; a few are depicted in Figure 11. If the required tag is specifically for flexible applications, then the tag selection parameter of flexible materials will be opted for. Further, thickness is considered as it should be feasible for the tag to be deployed in the required application. Cost is also an important factor that must be considered. Application-specific tags are designed depending upon the need of the application; the materials and parameters are selected based on this.

High reliability and sufficient thermal electrical performance are the main requirements of substrates to be deployed in electronic devices. In addition to the inherent material properties, the fabrication process also plays a critical role. In RFID tags and antennas, the substrate serves as the structural foundation that integrates and supports all functional components. The radiating element is typically either etched or printed onto the substrate surface. Substrate materials are generally categorized as either rigid or flexible. The selection of appropriate substrate material is influenced by various factors, including its mechanical and electrical characteristics, durability, resistance to harsh environmental conditions, and its flexibility, particularly for applications requiring conformance to non-planar or flexible surfaces [34]. Advancements in materials to be used as substrates is one of the areas of focus in research today. A high output along with reliability are driving the demand for the evolution of materials. Table 1 provides a general view of the characteristics of substrate materials. The dielectric substrate has a substantial impact on the spectral signatures of chipless RFID tags. This influence stems from various factors, including effective permittivity, radiation loss, conduction loss, and dielectric loss. Notably, effective permittivity significantly shapes the tag’s behavior, irrespective of environmental variations. While radiation loss is advantageous, minimizing conduction and dielectric losses remains crucial for achieving optimal performance [35].

This section illustrates multiple materials deployable as substrate materials: (i) rigid materials, (ii) flexible materials, (iii) 3D-printable materials, and (iv) advanced substrate materials.

### 3.1. Rigid Materials

Hard conventional substrates come under this first category of materials to be deployed as substrates. These rigid materials have been extensively used for chipless RFID tag designs and antenna designs for multiple applications. Even with the advancements of new materials, these typical materials have not lost their worth for technology. Hard materials are still under extensive deployment because of need, cost-related concerns, and ease of fabrication. FR4, Taconic TLX-0, and Rogers RT/duroid 5870 are some commonly used substrate materials in the identification and wireless communication domain to be used in RF identification tags and antennas. Rigid substrates are one of the top choices for the point of purchase (POP) if the focus is to establish something customized and lightweight.

Diverse research has been performed via utilization of rigid substrates not only for identification but also in the domain of sensing units for IoT systems. A tabular characteristic analysis of a few research outputs from using such conventional materials as substrates in antennas and the RFID domain is shown in Table 2 and Table 3.

### 3.2. Flexible Materials

One of the key pillars of flexible electronics for the future wireless era is the utilization and deployment of flexible materials. Flexible materials are extensively employed by researchers to come up with chipless RFID tags for traceability and sensing. Flexible materials are extensively used in wearable applications, RFID chipless sensors, and smart watches [33]. The flexible chipless RFID tag’s radiator pattern is made on its flexible substrate. Depending upon the planned application, flexible material is considered while analyzing the challenges faced compared to rigid substrates. Less weight, high thermal stability, deformability, severe environmental conditions, and complicated fabrication processes are a few issues to be faced when opting for flexible materials. Some basic requirements for flexible materials to be used as substrates are a high elastic modulus and electrical insulation, whereas the dissipation factor needs to be lower. On the other hand, the material’s stiffness, moisture absorption, weight, fabrication complexity, and cost must be lower [3].

The substrate material not only holds the radiator but also provides flexibility to the tag/antenna. This flexible nature is utilized for a wide range of applications, ranging from smart tags, wearable items, military, telemedicine, and health monitoring devices to aeronautics [48,49,50,51,52,53,54,55,56,57,58]. Various flexible substrate materials are shown in Table 4. Extensive research has been performed utilizing the flexible nature of paper [59,60,61,62,63,64], textile materials [65,66,67,68,69,70], and polymers [71,72,73,74,75,76,77,78,79,80,81,82,83,84,85,86,87,88,89] in the antenna domain.

#### 3.2.1. Paper as Flexible Substrate

One of the best environmentally friendly substrate materials is paper, which is extensively used in green electronics-based tags/antennas. Its low cost and non-complex printing aspects make the substrate outstanding among other high-cost materials which require extremely complicated fabrication/printing. Paper’s flexible nature is acquired from wood sources. Along with substrate utilization, paper is also used as an active material to take advantage of its properties [97].

#### 3.2.2. Textile as Flexible Substrate

Textiles are used as a substrate material for wearable applications, along with conductive threads to utilize their best properties. One of the major characteristics that matters when using textile materials is their loss tangent (Tanδ) and relative permittivity (Ɛr) values. These depend upon the thickness of the substrate/woven textile material or the number of threads/layers deployed [98]. Silk, cotton, wool, felt, and viscose are commonly used textile materials [99]. Moreover, to acquire high flexibility and conductivity, the materials are deployed with conductive polymers. Knitting, weaving, tufting, and nonwoven techniques are used for formation of textile substrates [100]. A promising application of textile materials is their utilization as green nanomaterials, primarily due to their sensitivity to solar radiation. Their capacity to absorb, transmit, and reflect solar rays makes them particularly well suited for use in ultraviolet (UV) protective textiles. Specific nanomaterials, such as titanium dioxide (TiO_2_), zinc oxide (ZnO), and silver nanoparticles, are commonly incorporated into textiles to enhance UV shielding properties. Additionally, coatings like silica-based nanoparticles and graphene oxide are increasingly utilized to improve the UV resistance and durability of textiles without compromising their flexibility and comfort [101].

Textile substrates can be used for a wide range of applications via manipulation of versatile parameters/properties, i.e., adsorption, adhesion, wettability, friction, and biocompatible nature. These factors govern the material’s performance, functions, and potential applications [102]. One of the high-output-yielding applications is surface activation of textile materials. This process, also known as ‘surface modification’, varies the surface properties of textile materials among other processes, roughening, etching, or ablation [103].

#### 3.2.3. Polymers as Flexible Substrate

**Figure 12:**
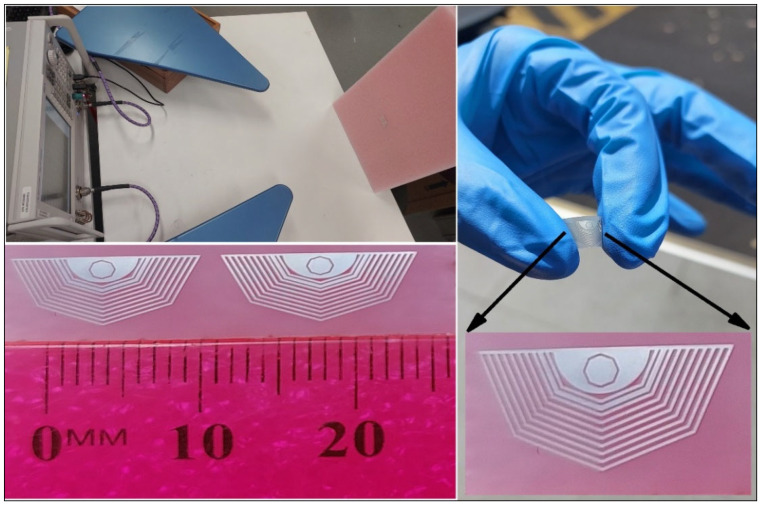
Polyethylene terephthalate (PET)-based tag and experimental setup.

One of the good choices for flexible materials to be used as substrates for RFID technology is polymers. Polymers are classified into natural and synthetic polymers depending upon their composition. Polymers are nonconductive materials that have a very highly resistant nature. Starch, wool, rubber, and silk come under the natural polymer category. Whereas PI (polyimide), PET (polyethylene terephthalate), PDMS (polydimethylsiloxane), PTFE (polytetrafluoroethylene), and PVC (polyvinyl chloride) are synthetic polymers, having a very highly flexible nature, tolerance to high temperatures, a low cost, and low thickness [33]. Figure 12 shows our lab experimental setup designed for PET-fabricated chipless tags. Alongside it, a flexible, bendable single unit and an array tag are shown.

**Figure 13:**
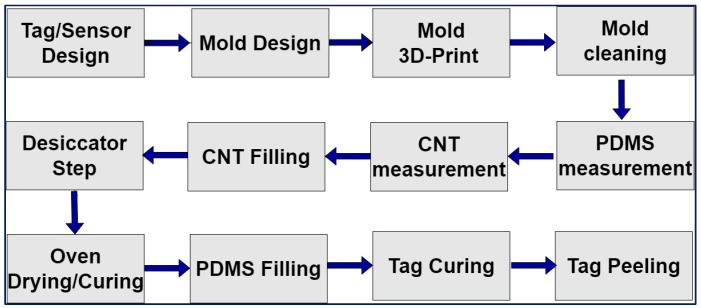
Fabrication steps of PDMS tag.

**Figure 14:**
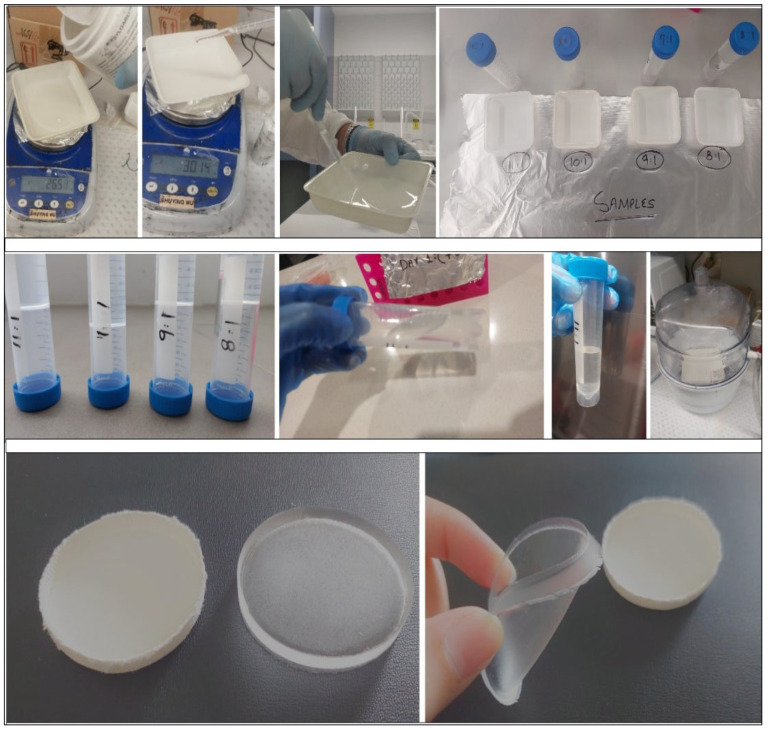
PDMS tag fabrication in laboratory.

Polydimethylsiloxane (PDMS) is a mineral organic polymer, commonly known as silicone. It is in the siloxane family, containing carbon and silicone. PDMS liquid has the benefit of having a low molecular weight. When the molecular weight increases, PDMS adopts a rubber/resin like texture. Having a dielectric constant between 2.3 and 2.8, the transparent, flowable, and water-resistant material exhibits outstanding chemical properties and low electrical conductivity [104,105]. The properties of this stretchable material can be altered depending upon customized application via doping with other substances. The flexible nature of PDMS also has some negative features, with it having a high cost and complicated fabrication process in comparison to other stretchable materials. The flexible nature of the material means it is used in various applications [106,107]. The flow of the fabrication process of PDMS is illustrated in Figure 13. Also, Figure 14 illustrates experimentation and self-fabrication of chipless RFID tags for 3-bit response measured for varying ratios of PDMS. Herein, we have demonstrated measurement of varying ratios for PDMS fabrication: 8:1, 9:1, 10:1, and 11:1. Then, the samples were cured. Also, a circular mold along with a cured, flexible PDMS sample is shown. The PDMS/CNT-based tag fabrication process involves design of the tag, mold 3D printing, preparation of PDMS in the correct ratio with a curing agent, CNT mixing, filling of the mold with CNT, and curing in an oven for 8 h; then, PDMS is poured on top followed by curing again for 10 h. At the end, the sample is peeled off to acquire the fabricated tag.

Lightweight plastic polyethylene terephthalate (PET), also named PETE, is a flexible material commonly referred to as polyester. PET, having extraordinary physical and chemical resistive properties, stays in a semi-crystalline state and has a transparent texture as well. The flexible material is widely deployed in a wide range of applications for chipless RFID tags [108,109,110], recycling applications, flexible antenna design [111], and antennas designed for 5G applications [112].

Liquid crystal polymer (LCP) is a special thermoplastic material which can stay in both the liquid amorphous/melted form and the crystalline solid state as well. It is a robust material because it withstands extreme temperatures, weather, and chemical exposure [113]. The polymer is used in applications where chemical and high-temperature resistance are required. Its high cost and complex fabrication are hindrances in utilization of LCP. Even then, the material is widely used in flexible antenna designs [114,115,116], bending-effect analysis in antennas using LCP [117], and is also deployed for WiMAX and WLAN applications [118].

Polyimide (PI), a temperature-stable polymer, can be found in two states: thermoplastic and thermosetting. The polymer demonstrates the potential to replace metals, steel, and glass in a variety of application areas. It is particularly well suited for high-temperature environments. The material’s chemical structure consists of two acyl groups bonded to nitrogen. However, its high manufacturing costs and the elevated temperatures required for processing remain significant drawbacks [119]. Antenna/tag substrates, smart electronics, and mobile phones are a few application domains of PIs. One of the extensively used polyimides is Kapton, broadly deployed in identification and the antenna domain [120,121,122,123].

Teflon is a highly flexible fluoropolymer of tetrafluoroethylene, referred to as polytetrafluoroethylene (PTFE). The material has amazing non-stick behavior. PTFE is also well known for temperature-resistant properties, as it offers extreme resistance to melting even at extreme temperatures. Also, the polymer repels oil and humidity. Researchers are always keen to utilize the astounding properties of the polymer in the antenna design domain [124]. Rogers Duroid is a commonly used PTFE type for chipless RFID tags.

### 3.3. 3D-Printable Materials

The 3D printing industry is booming, and future advancements are under way in the utilization of techniques in various industrial areas. To cope with the demands of printing, 3D printing machines are also flourishing. Nylon, acrylonitrile butadiene styrene (ABS), resin, polylactic acid (PLA), gold and silver, stainless steel, titanium, ceramics, PET/PETG, high-impact polystyrene (HIPS) are used in the 3D printing process [125]. Plastics classified as thermoplastics and thermosetting plastics can be used for printing via any of three 3D printing processes [126]:

- Stereolithography (SLA) 3D printers;
- Fused deposition modeling (FDM) 3D printers;
- Selective laser sintering (SLS) 3D printers.

FDM is low-cost but less accurate. SLA offers high accuracy, is suitable for versatile materials, but is sensitive towards UV radiation. SLS offers strong functional components but has very costly hardware. Table 5 outlines 3D-printable materials’ properties along with their application domains.

### 3.4. Advanced Substrates

Metamaterials classified as non-homogeneous and liquid-based materials come under the category of advanced materials to be used as substrates. These materials have an immense impact on the antenna and RFID industry as they can yield strong electromagnetic designs via controlling and balancing their properties. The antenna bandwidth can be enhanced via use of non-homogeneous substrates [127]. Overall antenna performance can also be enhanced via embedding materials [128]. The concept of advanced materials lies in the strategy of using artificially synthesized metamaterials instead of using traditional materials. The bandwidth of antenna can be enhanced from 10.5% to 20.6% (−10 dB) via controlling the material properties through geometric structure tunning and constituent-element positioning [129]. In [130], an antenna is designed and optimized using meta-substrates, resulting in significant gain enhancement and efficient bandwidth utilization. However, the inherent inhomogeneity of these materials poses challenges in their manufacturing processes. Research using advanced materials is presented in Table 6.

## 4. Radiator Material

The selection of the radiator generally depends upon the tag/antenna design, the fabrication process, and the application. Diverse conductive materials are utilized as radiators in chipless RFID tags. With advancements in identification technologies, there is a need for smart materials for advanced IoT networks. In RFID tags, generally two materials are used for tag design. One is the conducting material, and the other is the substrate. So, the radiator should be in accordance with the chosen substrate material for proper output and an appropriate fabrication technique. The main fabrication processes are etching and printing techniques. Chemicals are used in the former that sometimes generate waste. To overcome that drawback, advanced inks can be used in printing processes [134].

The radiator materials are classified into five categories: conventional metals, flexible materials, stretchable materials, ink-based materials, and advanced materials. For flexible tags/antennas, to come up with a flexible identification transponder the conductive material should be selected according to the flexible substrate being used. A comparison of the characteristics of radiating materials is listed in Table 7. The most common radiators used are pure copper metal, intrinsic metals like silver and aluminum, and printable conductive inks, i.e., silver nanoparticles.

With advancements in technologies and the fast evolution of identification and sensing technologies, researchers are focusing on utilizing advanced chemical inks and nano-materials for smart identification [135]. In [136,137], it was declared that animal or human tracking can be achieved successfully via utilization of smart invisible RFID ink that acts as a chipless RF identification tag. Moreover, instead of using conventional copper metal, innovative copper ink is utilized in the RFID antenna tag designed by Kim et al., which is an example of radiator material advancements [138]. Enhanced output performance is yielded compared to traditional etched-copper radiators. Also, to avoid signal interference with metal objects, RFID on metal (ROM) is also used in weapon and tool tracking [139].

In the context of chipless RFID, the inclusion of resonators introduces additional factors related to material losses. Conduction losses, dielectric losses, and surface losses of metals have a substantial impact on overall system performance. These losses influence the behavior of the resonators, their spectral signatures, and consequently the effectiveness of the RFID system. Surface losses play a significant role in the performance of chipless RFID systems. Minimizing these losses through careful material selection and design optimization is essential for ensuring efficient and reliable RFID operation. Metals with higher conductivity, such as copper and silver, exhibit lower conduction losses, while metals with lower conductivity, such as aluminum, incur greater losses. The surface losses alter the behavior of resonators. For instance, in frequency-selective surfaces (FSSs) or metamaterial-based chipless tags, the resonant behavior depends on the metal’s surface properties. Surface losses modify the resonant frequency, bandwidth, and quality factor of these structures. The absorption and scattering characteristics of chipless RFID tags are influenced by surface losses. As the metal absorbs energy, it affects the spectral response of the tag. This can lead to shifts in resonant peaks, changes in reflection coefficients, and altered readout performance. Surface losses impact the read range of chipless RFID tags. Excessive losses reduce the effective read range because less energy is available for backscattering. Additionally, sensitivity to incident power levels may vary based on surface loss effects. Achieving a balance between minimizing losses and maintaining other performance metrics is essential. Material selection, surface treatment, structural optimization, frequency-band selection, antenna matching, and testing are a few ways to balance these tradeoffs.

This section illustrates materials deployable as radiator materials in antennas and chipless RFID tags: (i) conventional metals, (ii) flexible metals, (iii) stretchable metals, (iv) ink-based metals, and (v) advanced radiator materials.

### 4.1. Conventional Metals

There are diverse papers stating coding techniques and various chipless tag designs [140], but the major topic of selection of an appropriate material is left blank. Copper, silver, and aluminum metals are conventionally used as radiator materials in tag/antenna designs. The most used radiator, along with various hard substrates, is copper metal. In [45], the authors deployed copper as the conductive part of the chipless RFID tags. In [141], Savill and Jewell used zinc metal with a thickness of 0.04 mm, along with steel and some other dielectric materials, to analyze the enhanced performance of organic coatings. Aluminum alloy has extensive application in the aerospace industry [142]. In [143], a metal–insulator–insulator–metal (MIIM)-based rectenna is presented that shows excellent efficiency, and the utilization of various metallic conductors is outlined, i.e., aluminum, copper, gold, and silver, which are ultimately deployed in energy-harvesting domains. Moreover, metal conductors have great potential for deployment as electrodes for electronically rewritable chip-free RF identification tags [144]. Table 8 presents the utilization of some conventional metals in various research articles.

Other metals that are used mostly as antenna conducting materials are annealed copper, gold, calcium, tungsten, zinc, nickel, and iron [150]. A material is selected via looking at its ability to conduct electricity, its oxidation resistance, cost, and structural integrity and stability. Silver leads the other mentioned metals in conductivity, whereas iron is at the bottom of the list. Even then, being cheap, iron is very commonly deployed in indoor antennas. Looking at oxidation, as the metal interacts with air a chemical reaction occurs, and it is oxidized. As a result of oxidation, the conductivity of metals decreases, and in return the antenna efficiency is decreased. Gold is the best choice of metal in this regard, as it rarely oxidizes. Lastly, analyzing the cost aspect of the materials used as conducting metals in antennas, the best choice is iron because of its low cost and high availability [150]. So, we can conclude that gold is the most suitable metal because of good conductivity and resistance to oxidation but it is very expensive to deploy it for all applications. Looking at the cost issue, iron is the best deployable metal but again it has very low conductivity and an issue of extreme oxidation. So, we can conclude that if the elements are covered by a plastic coating to avoid oxidation, then silver is very suitable for its easy and efficient deployment in antennas because of its very high conductivity.

### 4.2. Flexible Radiating Materials

All bendable metals and conventional metal tapes, such as silver tape, aluminum tape, and copper tape, fall under the category of flexible metals used as radiators. Rigid yet bendable metals can be utilized as metallic tapes in the design of antennas and tags for flexible applications. Copper tape is commonly employed in the fabrication of RFID tags and antenna prototypes, for establishing ground-plane connections, and for covering cracks [151]. Multiple copper tape layers can be deployed to enhance the shielding effect. In [152], copper cladding was used with the FR-4 substrate to acquire biomass. In [153], the authors showed that resonance frequencies can be improved if copper tape is deployed as the conducting material in the antenna design and simultaneously wave propagation is also tuned. Also, Nakamura and Hirayama reported that the conduction loss is reduced via deployment of copper tape in antenna design, and hence the Q factor of the antenna is enhanced [154]. Aluminum tape/sheet has a broad application for Tetra Pak package structures [155]. Copper tape along with aluminum foil is used to make indoor HF foil antennas [156]. Flexible metals’ utilization in antenna designs is shown in Table 9.

### 4.3. Stretchable Radiators

Fabric- and thread-based textiles used as radiators come under the stretchable metals category. Apart from flexible materials, stretchable materials are also deployed in antennas/RFID tags for flexible applications in wireless and multi-identification/sensing domains. Using fabric composites not only adds flexibility to the antenna/tag but also offers biocompatibility and uncomplicated fabrication methods [160]. Polymer threads and conductive metals comprising conductive threads yield E-textiles (conductive textiles). These smart E-textiles can yield flexible antennas/tags that can be deployed directly on smart garments. Nickel–copper fabric, Zelt, Pure Copper Taffeta Fabric, and Shieldit Super are a few fabrics commonly used as radiating antenna parts [161]. Embroidered conductive fabrics have the capability to yield flexible antennas with enhanced robustness and durability because of E-Threads’ firmness [162]. Embroidered tags seek easiness of embedding into any cloth/fabric instead of complicated fabricated techniques. One major issue these flexible tags face is radiation degradation because of twisting/bending of fabric. In the case of antennas, gains, directivity, and radiation patterns are the possible affected parameters. The tags/antennas should analyze the bending effects to provide a tolerance level for twisting/bending effects, especially in the case of RFID tags as the bending can cause variation in resonant radiators, hence shift/alter the RCS response [3]. In [3], the author studied bending effects on two flexible chipless RFID tags.

Furthermore, smart E-Thread technology is gaining significant traction in the realm of identification, positioning itself as a key technology for the future of IoT and AI systems. The thread is integrated into the fabric during the manufacturing process, thereby eliminating the need for additional embedding time and reducing associated costs. Additionally, the smart fabric provides automatic protection against theft, as the embroidered thread cannot be easily identified or removed for malicious purposes. While the E-Thread tag offers a long lifespan and high durability, its primary drawback remains its higher cost in comparison to conventional UHF RFID tags [163]. The stretchable fabrics/materials that have been deployed as antenna and tag radiating parts are depicted in Table 10 and Table 11 with some used cases.

Along with E-Thread radiators, the fabric substrate plays an important role in the antenna gain. Curtain cotton, wash cotton, jeans, and polycotton are fabric substrates that are extensively used in E-Thread antenna designs. Annalakshmi et al. have designed a flexible E-Thread antenna using jeans, Teflon with copper E-Thread, and one antenna comprising a stainless-steel E-Thread embroidered on a cotton substrate [165]. The former was made using a printing technique for fabrication, while the latter used a hand-sewable stitching fabrication process.

### 4.4. Ink-Based Tags/Antennas

Printing techniques have been revolutionized by fabrication using conductive ink. Printing now takes less time compared to traditional etching fabrications. Also, it allows for layer formations for the conductive part of the antenna/tag, i.e., additivity, whereas etching is a subtractive fabrication method. Graphene, aluminum, and silver inks are the most deployed conductive materials for printing. Conductive inks are prepared by mixing different ratios of the various components to be deployed on the substrate. Offset, flexo, gravure, screen, and inkjet printing are used in various domains [170]. Long-term stability, environmental friendliness, adhesion to the substrate, optical transparency, low-cost, electrical properties, and suitable dispersion are some of the most important properties required of conductive inks [171].

**Figure 15:**
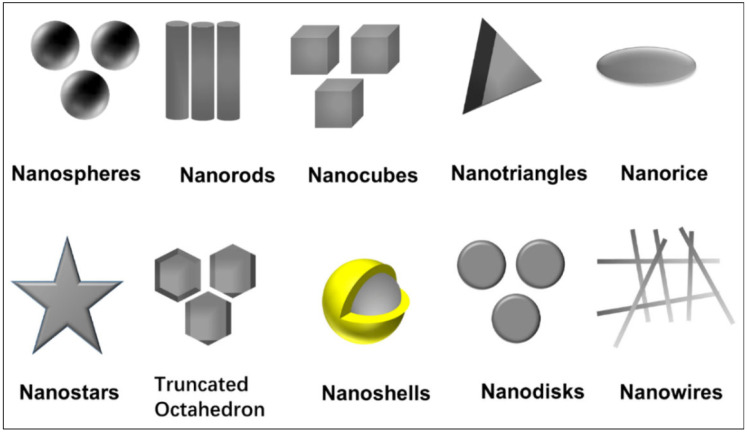
The various shapes of silver nanoparticles [174].

Poor conductivity and harsh environments are two major points of concern. In [172], a comparative study of silver ink, copper, and aluminum dipole antennas was conducted to analyze their performance with clad copper and etched copper, offering insights into the advantages of conductive inks. The authors conclude that copper and silver nanoparticle-based inks hold significant potential. High gains, essential for wireless applications, can be achieved through high-conductivity radiators. Notably, deposited copper exhibits much higher conductivity compared to silver ink [172]. Silver and copper nanoparticle inks are recommended for flexible antenna/tag designs because of their high conductivity [173]. Because of having a low risk of chemical oxidation reactions, silver nanoparticles have an edge over copper nano-ink for flexible applications. Graphene finds its place in the flexible antenna domain because of its useful mechanical properties and fine conductivity. Graphene is used as an oxide ink, nanoflake ink, graphene paper, and nanoparticle-based ink for flexible wireless applications. Graphene-based antennas offer enhanced bandwidth compared to rigid antennas with copper metal. Table 12 and Table 13 show various research outcomes using conductive inks. Ref. [174] shows the various possibles shapes of silver nanoparticles; these are depicted in Figure 15.

### 4.5. Advanced Materials

Hybrid metals, CNT nanoparticles, and liquid crystals are leading to advanced materials in identification and wireless communication areas [182]. Hybrid metals are the outcome of different composition ratios of various metals and inks to acquire excellent characteristics for feasibility and deployment. Graphene is the target of researchers in hybrid metal making. Moreover, researchers have performed much research on CNT nanoparticles for THz components and energy harvesting [183]. CNTs are extensively flexible, very elastic, and have high electrical and thermal conductivity [184]. They are further classified as MWCNTs (multi-walled carbon nanotubes) and SWCNTs (single-walled carbon nanotubes).

Hajjyahya et al. illustrate a novel approach towards advancing materials by using coated carbon nanotubes in the nanoscale range [185]. New hybrid materials (SWCNTs-copper) and (SWCNTs-aluminum) are proposed by coating thin layers of copper and aluminum on SWCNTs for terahertz antennas. Graphene nanotubes (TUBALLTM) is a revolutionary material offering amazing properties with other additives, suitable for multiple applications ranging from sensors, electronics, automotive, and biomedicine to aerospace and aviation [186]. Hybrid carbon nanotubes, doped with boron/nitrogen and coated with gold plating, can achieve significantly enhanced conductivity [187]. In [188], the authors employed a PDMS substrate integrated with CNTs to create a PDMS-CNT composite fiber, which was then utilized as a strain sensor. Additionally, researchers are increasingly focusing on the use of metamaterials in this field [189]; one such work is based on the design of a five-layered dielectric resonator antenna based on hyperbolic metamaterials [190]. This HMM design is based on a silica glass substrate with a ZnS layer, nano-diamond crystal, and metallic dielectric layers, and is referred to as a five-layered Ag/Au-ZnS resonator. Using additive manufacturing (AM), researchers are keen to come up with advanced 3D-pritnable multifunctional composites [191]. CNTs and graphene can be used with polymeric ink to develop such CNT–polymer or graphene–polymer composites for sensing applications. Some characteristic uses of advanced materials are shown in Table 14.

Apart from all these materials, an advanced material derived from carbon is capturing researchers’ attention for deployment in nano-technology antennas. MXene (Ti3C2Tx) is a flexible advanced ink material having applications in sensing and 5G applications [196,197,198,199,200,201,202]. Its high electrical conductivity, excellent electromagnetic interference shielding, high electrical conductivity and tunable surface chemistry, large specific surface area and excellent hydrophilicity, and affordable synthesis are a few of the important aspects of Mxene that makes it very useful in RFID tag applications. Moreover, liquid crystals (LCs) are a focus of researchers now because of their performance. Liquid crystals have a low cost and require low power, with upgraded performance parameters. Smart antennas based upon liquid crystals (LCs) are the focus of research these days. LCs are a part of TV and mobile smart phone screens. Previously, LCs have been extensively used in satellite communication because of their high availability and low cost. Liquid crystals add stretchability to antennas, so they are of great benefit for use in stretchable antennas and conductors [203]. Researchers are also looking to use liquid crystals as a substrate because of their varying permittivity properties that allow them to be used as a tunable dielectric substrate in antennas [204]. Such liquid crystals find application in mm-W communication, offering smart antenna solutions [205]. A few initial research works on liquid crystals in the antenna domain are shown in Table 15.

Development of advanced materials is the main driving force for the evolution of chipless RFID technology. (i) Cost reduction: Traditional RFID tags with chips can be expensive, particularly in large-scale applications. Materials that allow for chipless designs help lower manufacturing costs significantly. For example, using printed conductive inks or flexible polymers reduces the need for expensive silicon chips. (ii) Miniaturization and flexibility: Advanced materials, especially those like metamaterials or flexible conductive polymers, enable the creation of smaller and more flexible RFID tags that can be used in a wider range of applications. These tags can be integrated into items where traditional, rigid RFID chips would not work (e.g., clothing, food packaging, medical devices). (iii) Scalability: Chipless RFID offers the potential for highly scalable production. Using advanced materials like printable inks and nanomaterials, manufacturers can produce large volumes of tags at a lower cost, making RFID more accessible for consumer goods and logistics companies. (iv) Environmental adaptability: Advanced materials can be tailored to perform well under a variety of environmental conditions, such as extreme temperatures, humidity, or exposure to chemicals. This makes chipless RFID tags more suitable for industrial, agricultural, and medical applications, where traditional RFID tags might fail. (v) Smart and secure tagging: The materials used in chipless RFID can also help improve the security of RFID systems. For example, the unique frequency signatures created by certain metamaterials can make it harder to clone or counterfeit RFID tags, providing enhanced security for applications like inventory tracking or authentication systems. A comparison of flexible and non-flexible antenna designs, and RFID tags is shown in Table 16. The table provides a comparative overview of various antenna designs and RFID tags, highlighting the materials used, their flexibility, and performance characteristics. It serves as a valuable resource for selecting appropriate antenna designs for specific RFID applications.

Materials are chosen based on the specific application requirements and classified as either rigid or flexible. The development sequence of the selected materials is outlined, including a rationale for each material choice, with a focus on their intrinsic properties. Key factors considered include performance, electrical properties (such as permittivity, loss tangent, conductivity, and resistivity), material cost, and availability. The fabrication process for any unique tag or antenna design is also critical. These factors are interdependent, with decisions made by the intended user based on their specific needs. These considerations drive improvements in key RFID system parameters, such as read range, miniaturization, and environmental adaptability. This addition aims to clearly demonstrate how material selection directly impacts system-level performance enhancements.

## 5. Conclusions

With the advancement of technologies and upgraded hybrid material combinations, conventional metals and substrates have not lost their importance and continue to be utilized in applications ranging from identification and wireless domains [215,216,217] to smart building glass antennas and energy-harvesting applications [218,219] in smart wireless systems. Presently, researchers are keen to come up with man-made/altered materials known as metamaterials and nanomaterials [220]. These materials improve performance, which results in efficient high-gain antennas [221]. Moreover, recently graphene nanotubes have been made for commercial use for the first time. The material, also referred to as single-wall carbon NTs, is used to enhance the physical properties of other materials via additivity [222]. With emerging materials, conventional metals and substrates have not lost their utilization. A cancer-detecting antenna composed of the FR4 substrate along with copper metal is an example of such an application [223]. Moreover, instead of copper metal or tape, utilization of copper paste for antioxidant flexible wearable electronic devices has addressed the need for high-conductivity stretchable systems [224]. To overcome the tradeoff between bandwidth, gain, and antenna/RFID tag size, metamaterials with properties that yield enhanced bandwidth can be deployed for miniaturized antennas [225]. Also, metamaterials, if deployed on innovative on-chip antennas, not only reduce substrate losses but also improve bandwidth and antenna gains [226]. All the materials, either conventional or advanced, find extensive applications in 5G and 6G wireless communication [227,228].

The printed electronics domain can be revolutionized if we replace high-cost, non-environmentally friendly metallic inks, conductive polymer inks, and carbon-based inks with graphene ink, which has impeccable characteristics. The potential of the highly electrically conductive graphene is very useful for flexible electronics but there is a need to overcome challenges related to its stability and water dispersion. Technology can be upgraded by the creation of hybrid conductive inks via formulations involving graphene and conductive polymers or metal nanoparticles. The need of the era is to redefine future technologies via using extensively conductive inks and nanowires such as graphene as suitable substitutes for identification and wireless communication applications [229]. Lastly, more effort and research are required in the future to evolve smart E-Thread technology to allow IoT to embrace new images across the globe. Durable RFID solutions embedded/threaded in cloth will not only manage tracking and logistics but will also have the capability to consider customer returns, secondhand products, and the recycling stage of fabrics. More outcomes can be yielded by using advanced MXene ink and liquid crystal radiating materials for wireless and sensing applications. Materials such as ‘MXene ink’ are crucial in the manufacturing process of these tags. Utilizing these materials could potentially lower the cost of production, enhance the coding capacity, and allow for the reconfiguration of ID generating circuits, among other benefits. Ultimately, it could be a significant step towards designing smart tags for smart wireless networks. Cutting-edge MXene ink and liquid crystal radiating substances can yield effective outcomes for wireless and sensor-based applications. MXenes have demonstrated significant promise in a range of applications, notably in chipless technology [230]. MXenes are recognized for their outstanding electrical conductivity, a key characteristic for chipless technology. Efficient signal transmission and processing, crucial for the performance of chipless devices, is guaranteed by their high conductivity [231]. Also, the number of M-X-M layers in MXenes can be varied, allowing for a significant level of structural manipulation. This adaptability can be utilized to customize the characteristics of chipless devices to meet specific needs. MXenes have demonstrated their potential as fillers in the creation of multifunctional stress sensors, usable in chipless RFID technology. The distinct characteristics of MXenes, such as their exceptional conductivity, adaptable structure, multiple functionalities, scalability, and open framework, position them as a potential candidate for integration into chipless technology. Nonetheless, additional exploration and advancement are required to completely utilize their capabilities in this domain [232,233,234].

Chipless RFID technology is being propelled forward by the development of advanced materials that allow for cheaper, more flexible, and highly functional RFID tags. Metamaterials, conductive polymers, carbon nanotubes, and printed electronics are central to driving this progress. The core driving forces of these materials are cost reduction, scalability, flexibility, miniaturization, and security, which collectively open new use cases and drive the adoption of RFID across industries.

Printing techniques should be upgraded to incorporate high-resolution and enhanced performance, as up till now, the direct printing of highly electrically conductive materials is still a challenge [235]. Also, regarding conductive inks, adequate formulations must be achieved for useful outcomes. Efforts are required to simplify the fabrication process of conductive inks. To make the ink technology green, the use of toxic solvents in the fabrication process should be altered. These factors will lead to acquiring the best printed flexible wearable electronics. To visualize the dreamed of 6G and beyond networks, artificial intelligence (AI), along with the smart IoT, can come up with high data rates, miniaturized electronics/antennas, energy efficient, and connected intelligent networks.

This article provides a comprehensive guide for the implementation of inexpensive passive chipless RFID tags in smart wireless networks, utilizing recent advancements in the Internet of Things (IoT) and AI-enabled IoT networks. The research will be expanded to explore the relationship between the choice of materials and their impact on cost reduction, manufacturing duration and methods, expenditure, and technological progress. Also, work needs to be performed to streamline the manufacturing process of conductive inks. To make ink technology more environmentally friendly, we should consider changing the harmful solvents used in fabrication to safer alternatives.

## Figures and Tables

**Table 1:** Characteristics of substrate materials.

Materials	Rel. Permittivity	Loss Tangent	Flexibility
FR4	4.3	0.025	x
Taconic TLX-0	2.45	0.0019	x
Taconic TLX-8	2.55	0.0019	x
Rogers RT/duroid 5870	2.33	0.0009	x
Rogers RT/duroid 5880	2.20	0.0009	√
Thermoset Polyester	4.0	0.0050	x
Kapton^®^ HN	3.5	0.0026	√
PDMS	2.76–3.00	0.01–0.05	√
SYLGARD™ 527 Silicone Dielectric Gel	2.85	0.0001	√
SYLGARD™ 170 Silicone Elastomer	2.5	0.0002	√
SYLGARD™ 182 Silicone Elastomer	2.65	0.0005	√
SYLGARD™ 184 Silicone Elastomer Kit	2.68	0.00133	√
PET	3	0.0025	√
HP Photopaper	3.2	0.04	√
Teflon (PTFE)	2.1	0.00015–0.0003	√
PE	2.25	0.0005	√
Parylene C	2.7	0.1	√
Parylene N	2.7	0.0006	√
PLA	3.11 ± 0.07	0.013 ± 0.001	x
ABS	2.0–3.5	0.00500–0.0190	x
Nylon	2.4	0.0083	√
Desmopan^®^ TPU	8.0–4.0	<0.02	√

**Table 2:** Characteristics of rigid materials used in antennas.

Ref #	Substrate	Permittivity/ Loss Tangent	Thickness (mm)	Oper. Freq./ Freq. Band (GHz)	Design	Area (mm^2^)	Gain (dBi)	Radiation Efficiency (%)	Application
[36]	FR-4 laminate	4.3 -	1.6	9.33 2.77–12	MIMO antenna	90 × 90	5	>75	UWB MIMO applications
[37]	FR-4	4.4 -	0.8	3.5/4.8	MIMO antenna	150 × 75	>2	55/72	5G Communication
[38]	Rogers RT/duroid 5880	2.2 -	0.787	8.5 3–12.7	UWB-Fractal antenna	24 × 30	3.6	88	Microwave imaging applications
[39]	-	10.2 -	0.762	10.5 9.2–10.1	CP MIMO antenna	35 × 30	6	-	X-band applications
[40]	Rogers RT/duroid 4003	3.55 0.0027	0.203	28	Linear antenna array	39.5 × 39.5	13.01	83.05	5G new radio (NR) comm.
[41]	FR-4	4.4 -	1.6	3.21–3.81	MIMO antenna	150 × 75	3.64	>90	5G mobile applications

**Table 3:** Characteristics of rigid materials used in RFID tags.

Ref #	Substrate	Thickness (mm)	Radiator	No. of Bits	Area (mm^2^)	Freq. Band (GHz)	Sensing	No. of Tagged Items	Application
[42]	FR4	1.6	Copper	10	23.8 × 17	2–4	x	1024	Identification
[43]	FR4	0.8	Copper	2	21 × 21	2–4	x	4	ISM band
[31]	FR4	0.5	Copper	25	27 × 12.5	4.5–12.5	x	33,554,432	Tracking
[44]	FR4	0.5	Copper	9	12.4 × 6	6–16	x	512	Pharmaceutical industries
[45]	Taconic TLX-0	0.635	Copper	13	16.65 × 17	3–12	x	8192	Item-level tagging
[44]	Taconic TLX-0	0.5	Copper	9	12.4 × 6	6–16	√	512	RFID sensing
[46]	Rogers-RT/duroid 5870	1.575	Copper	10	10.8 × 10.8	3.5–7.5	x	1024	IoT market
[47]	Rogers RO4003	0.88	Copper	12	29.75 × 34	3–7	√	4096	Temp. sensor

**Table 4:** Characteristics of flexible materials.

Ref #	Substrate	Thickness (mm)	Radiator	No. of Bits	Area (mm^2^)	Freq. Band (GHz)	Sensing	Application
[90]	Kapton HN	0.125	Silver nano-ink	27	22 × 22	3.7–15.1	x	Flexible identification
[91]	Kapton HN	0.125	Copper	22	171.9	4–25	√	Aviculture industry
[92]	Rogers RT/duroid 5880	0.508	Copper	38	29 × 29	4.7–14.8	x	Smart IoT applications
[93]	Rogers RT/duroid 5880	1.575	-	26	20 × 20	3.5–8	x	Item-level tracking
[94]	PET	0.1	Copper	9	-	4.7–13.7	x	IoT enabler
[3]	PET	0.07	Gold	8	25 × 25	4–18	x	
[95]	PDMS	0.4	Nickel–copper coated ripstop	6	25 × 25	3.1–10.6	x	WBAN IoT
[96]	HP Photopaper	0.25	Silver nano-ink	15	20 × 10	2.4–14.6	√	Smart green electronics

**Table 5:** Characteristics of 3D-printable materials.

No.	Material	Printing Technique	Characteristics	Utilization
1	ABS	FDM 3D Printing	Temp. resistant	Functional prototypes
2	PLA	FDM 3D Printing	Easy printing	Resemblance models
3	PETG	FDM 3D Printing	Moisture resistant	Moisture-resistant applications
4	Nylon	FDM 3D Printing	Complicated printing	Lightweight applications
5	TPU	FDM 3D Printing	Flexible	Stretchable prototypes
6	PVA	FDM 3D Printing	Water soluble	Assistant/holding material
7	HIPS	FDM 3D Printing	Soluble support	Aid/support material
8	Standard Resins	SLA 3D Printing	Even surface	Pretend prototypes
9	Clear Resins	SLA 3D Printing	Optical transparency	Millifluidics
10	Draft Resin	SLA 3D Printing	Rapid printing	Faster iterations
11	Tough Resins	SLA 3D Printing	Stretchable, durable	Connectors
12	Rigid Resins	SLA 3D Printing	Sustains the load	Automotive housing
13	High Temp. Resins	SLA 3D Printing	Elevated accuracy	Temperature resistant housings
14	Flexible Resins	SLA 3D Printing	Withstands bending/flexible	Medical/robotics applications
15	Medical Resins	SLA 3D Printing	Biocompatible	Medical/dental equipment
16	Ceramic Resins	SLA 3D Printing	Resembles stone	Art applications
17	Nylon 12	SLS 3D Printing	Temperature, moisture resistant	Medical instruments/gadgets
18	Nylon 11	SLS 3D Printing	Temperature, humidity resistant	Medical equipment
19	TPU	SLS 3D Printing	Deformation adaptable	Stretchable medical equipment
20	Nylon Composites	SLS 3D Printing	Strength	Effective prototyping

**Table 6:** Characteristics of advanced materials.

Ref #	Substrate	Operational Frequency (GHz)	Design	Dimensions (mm^2^)	Applications
[131]	Homogeneous	2.45	Patch antenna	35.9 × 3	Efficient miniaturization
[132]	Metamaterial	0.906	RFID tag antenna	60 × 19.89	High-efficiency
[133]	Metamaterial	2.4	RFID tag antenna	42.6 × 42.6	RF devices/optical devices

**Table 7:** Characteristics of radiator materials.

Material	Conductivity (Sm^−1^)	Resistivity (Ω-m)	Thermal Conductivity (W/m)	Density (g/cm^3^)	Printing Tech.
Copper	5.96 × 10^7^	1.7 × 10^−8^	401	8.9	Etching
Aluminum	3.56 × 10^7^	2.8 × 10^−8^	237	2.7	
Silver	9 × 10^6^	1.6 × 10^−8^	429	10.5	Printing
Graphene	1 × 10^5^	1 × 10^–8^	4.84 × 10^3^	2.267	-
HCGAF	1.82 × 10^6^	6.5 × 10^−8^	-	-	-
Gold	44.2 × 10^6^	2.4 × 10^−8^	317	19.4	-
MWCNT	1 × 10^5^	1.74 × 10^−8^	2586	2.3	-
SWCNT	10^2^ to 10^6^	5.34 × 10^3^	3000	1.8	-
MXene (Ti3C2Tx)	150	-	-	-	-

**Table 8:** Characteristics of conventional radiator materials.

Ref #	Radiator	Thickness (mm)	Substrate	No. of Bits	Area (mm^2^)	Freq. Band (GHz)	Sensing	Flexibility	Application
[43]	Copper		FR4	2	21 × 21	1–8	x	x	ISM band
[44]	Copper	0.035	Taconic TLX-0	9	12.4 × 6	6–16	√	x	Low-cost ID
[145]	Copper	0.035	Paper	30	-	22–26.5	x	√	Flexible detection
[146]	Copper	-	FR-4	12	35 × 33	3.1–10.6	x	x	Identification
[147]	Metallic	-	Rogers RO4350	20	60 × 60	3.1–3.9	x	x	Data-dense identification
[148]	Copper	0.035	Roger RT/duroid/5880	20	25 × 17	4.1–16	√	√	Conformal applications
[148]	Copper	0.035	Taconic TLX-0	20	25 × 17	3.8–15	x	x	Identification
[149]	Copper	0.012	PET	18	20 × 20	3.5–16	√	√	Biomass tracking
[3]	Gold	0.1	PET	8	25 × 25	4–12	x	√	Identification
[3]	Gold	0.1	PET	8	13.44 × 11.56	8–18	x	√	Identification

**Table 9:** Characteristics of flexible radiator materials.

Ref #	Radiator	Thickness (mm)	Substrate	Rel. Permittivity	Area (mm^2^)	Operating Freq. (GHz)	Freq. Band (GHz)	Applications
[153]	Copper tape	0.03	Kapton HN	3.5	65 × 46	2.4	-	ISM band
[157]	Copper tape	0.05	Denim jeans	1.54	46 × 16	2.45 5.8	-	ISM band Wearable applications
[158]	Copper tape	-	Cloth fabric	1.8	30 × 30	-	3.1–10.6	WBAN-UWB Biomedical applications
[159]	Copper tape	0.07	ABS	2.8	27.8 × 36.8	28	-	Commercial applications

**Table 10:** Characteristics of stretchable radiator materials.

Ref #	Radiator	Substrate	Thickness (mm)	Rel. Permittivity	Loss Tangent	No. of Bits	Area (mm^2^)	Freq. Band (GHz)	Sensing	Application
[1]	Nickel–copper fabric	PDMS	0.4	2.77	0.02–0.076	6	25 × 25	3.1–10.6	x	Wireless identification
[164]	Textile yarn	No substrate	-	2	-	-	135 × 0.44	0.864–0.867	√	E-Thread temp. sensor

**Table 11:** Characteristics of stretchable E-Thread radiator materials.

Ref #	Radiator	Conductivity (S/m)	Surface Resistance (Ω)	Substrate	Area (mm^2^)	Oper. Freq./Range (GHz)	Radiation Efficiency	Gain (dB)	S11 (dB)	Applications
[161]	Zelt Copper Taffeta Shieldit Super	1.479 × 105 2.5 × 105 6.67 × 105	0.05 0.05 1	Foam (3 mm, Ɛr = 1.006)	120 × 120	1.575	0.858 0.8962 0.978	6.735 7.564 7.7	−17.6 −14.5 −13.9	GPS applications
[162]	E-Threads	-	1.9	Felt		0.76–1.015	-		-	Flexible applications
[165]	Copper E-Threads	-		Cotton fabric	40 × 40	3.1–10.6	-	3	-	Military applications
[166]	Silver conductive thread/Rayon thread	-	0.70	Nylon (0.35 mm)	90 × 10	0.880–0.990	-		-	Smart wearable applications
[167]	E-Threads	-	1.9	Kevlar fabric (0.59 mm) (Ɛr = 2.6, Tanδ = 0.006)	160 × 160	0.3–3	-	6.5	-	Airborne and wearables
[168]	Elektrisola E-Threads	-	1.9	PDMS (1.5 mm) (Ɛr = 3, Tanδ <0.01)	160 × 160	1–5	-		-	Medical applications
[169]	Elektrisola E-Threads	-	1.9	PDMS (Ɛr = 3, Tanδ = 0.004)	160 × 160	1–6	-	6	-	Flexible applications

**Table 12:** Characteristics of ink-based conductive materials used in RFID.

Ref #	Radiator	Thickness (mm)	Substrate	No. of Bits	Area (mm^2^)	Freq. Band (GHz)	Sensing	Applications
[175]	Silver ink	0.015	Kapton HN	9	10.5 × 15	8–19	x	Ubiquitous sensor network
[175]	Silver ink	0.015	PET	9	10.5 × 15	8.5–20	x	Ubiquitous sensor network

**Table 13:** Characteristics of ink-based materials used in antennas.

Ref #	Radiator	Conductivity (s/m)	Substrate	Thickness (mm)	Dielectric Constant	Area (mm^2^)	Oper. Freq.	Freq. Band/ Band Width	Gain (dBi)	Applications
[164]	Graphene ink		FR4	1.6	4.4		2.45 GHz	2.421–2.474 GHz 74.5 MHz	0.94	ISM band
[176]	Silver ink	0.1–0.7	Sylgard 184 (PDMS)	3	3.0	60 × 20	2.50 GHz	- 105 MHz	-	Stretchable electronics
[177]	Silver ink	6.8 × 10^6^	PP foil	0.15	6.03	-	895 MHz	860–928 MHz -	2.8 1.5	Bluetooth Wi-Fi
[178]	Graphene ink	2.5 × 10^4^	Kapton HN	0.125	3.5	30 × 20	5.65 GHz	3.5–6.5 GHz 3 GHz	-	WLAN 5G appl.
[179]	Graphene ink	-	Teflon	0.8	2.65	25 × 15	-	2.83–6 GHz	−1.7	Flexible, printable electronics
[180]	Graphene ink	-	Paper	-	-	43 × 3	2.4 GHz	2.297–2.510 GHz	0.7	IoT sensing
[181]	Graphene ink	10^8^	Polyimide	0.016	4.3	0.62 × 0.8	7.5 THz	1–30 THz 10.96 THz	7	Terahertz communications

**Table 14:** Characteristics of advanced radiator materials.

Ref #	Radiator	Thickness (mm)	Substrate	Dielectric Constant	Loss Tangent	Area (mm^2^)	Freq. Band /Op. Freq.	Sensing	Application
[183]	CNT film	0.0014	Polyimide film	-	-	0.35 × 0.35	1.5 THz 361 THz	√	THz sensing
[192]	MWCNTs	0.5	PDMS	2.76	0.01	110 × 78	-	√	Water quality monitoring
[193]	SWCNT	0.032	Polyamide membrane	-	-	80 × 180	0.5–1.5 GHz	√	Ammonia sensor
[194]	Liquid metal (LM)	-	PDMS (Sylgard 184)	2.68	0.0375	30 × 12	2.4 GHz 5.8 GHz	x	Wrist-worn applications
[195]	Hybrid metamaterial	-	FR4	4.3	0.025	28 × 32	2.25–9.47 GHz	x	Multi-band wireless applications

**Table 15:** Characteristics of advanced LC (liquid crystal) materials.

Ref #	Radiator	Conductivity (S/m)	Thickness (mm)	Antenna	Substrate	Area (mm^2^)	Oper. Freq./Range (GHz)	Refl. Coefficient/Gain (dBi)	Application
[206]	3D LM composite	8.1 × 10^5^–1.3 × 10^6^	-	Dipole	PDMS	72 × 40	1.55–0.45	−30	Wireless strain sensor
[207]	E7-LC		0.254	Leaky wave (LWA)	Rogers 4350B (ε = 3.48, Tanδ = 0.0037)	-	26–30	5.5	Flexible antennas
[208]	(a) HC12-Thread (b) LCP-(LIBERATOR 40)	5000	0.09 0.12	4-bit tag	Plain cotton (0.25)	25 × 25	8–18	-	Smart textile applications
[209]	LC	-	-	Antenna array	-	-	2.4	3.5	Antenna array systems
[210]	N-LC	-	-	Microstrip leaky wave antenna (MLWA)	Rogers RT5880 (0.254 mm, Ɛr = 2.2, Tanδ = 0.0009)	157.5 × 36	8.4–10.5	7.53	Satellite and radar communication
[211]	Liquid metal (LM)	-	-	IFA antenna	PDMS (4.5 mm, Ɛr = 2.68, Tanδ = 0.0375)	30 × 12	2.4 5.8	5.55 1.66	Wearable applications

**Table 16:** Comparison of flexible and non-flexible antenna designs and passive RFID tags.

Ref. No.	Antenna/Tag Geometry	Material Type	Substrate Material	Flexibility	Operating Frequency (GHz)	Gain (dBi)	Bandwidth (MHz)	Bits
[27]	Square slot	Silver ink	PET	Flexible	1.35–6.9	N/A	N/A	3
[27]	Square slot	CNT	PDMS	Flexible	1.35–6.9	N/A	N/A	3
[45]	C-shaped slot	Copper	Taconic TLX-0	Non-flexible	3–12	N/A	N/A	13
[64]	Square slot	Copper	Paper	Flexible	1–12	N/A	N/A	28
[212]	Meander line	Silver ink	Kapton	Flexible	0.868	1.66	5	N/A
[212]	Meander line	Silver ink	PET	Flexible	0.868	1.66	5	N/A
[213]	Dipole	Copper	FR4	Non-flexible	2.45	2.1	N/A	N/A
[214]	Loop resonator	Liquid metal	PDMS	Flexible	0.868	2.7	N/A	N/A
